# Incidental detection of cancers during population-based endoscopic gastric cancer screening in Japan

**DOI:** 10.1007/s10388-026-01238-8

**Published:** 2026-08-10

**Authors:** Kensuke Uraguchi, Kenta Hamada, Naomi Matsumoto, Toshiharu Mitsuhashi, Mizuo Ando, Kenji Fujimoto, Shunsaku Hayase, Takashi Yorifuji

**Affiliations:** 1 Department of Otolaryngology–Head and Neck Surgery, Dentistry and Pharmaceutical Sciences, Okayama University Graduate School of Medicine, 2-5-1 Shikata-cho, Kita-ku, Okayama, 700-8558 Japan; 2 Department of Practical Gastrointestinal Endoscopy, Okayama University, Okayama, Japan; 3 Department of Epidemiology, Dentistry and Pharmaceutical Sciences, Okayama University Graduate School of Medicine, Okayama, Japan; 4 Center for Innovative Clinical Medicine, Okayama University Hospital, Okayama, Japan; 5 Occupational Health Data Science Center, University of Occupational and Environmental Health, Kitakyushu, Japan

**Keywords:** Gastric cancer screening, Endoscopy, Radiography, Early detection of cancer, Esophageal neoplasms

## Abstract

**Background:**

Upper gastrointestinal endoscopy traverses the full upper aerodigestive tract, unlike radiography, potentially enabling incidental detection of non-gastric malignancies. However, its population-level incidental detection rate for non-gastric upper aerodigestive tract cancers has not been systematically quantified. The aim of this study was to compare endoscopic screening with radiography and quantify detection of non-gastric upper aerodigestive tract cancers in a population-based setting.

**Methods:**

This population-based cohort study was conducted by linking the Okayama City municipal gastric cancer screening registry with the Kokuho Database (KDB) for fiscal years 2016–2021. Among 36,326 participants contributing 64,822 screening examinations (40,832 radiography; 23,990 endoscopy), diagnoses of oral cavity, pharyngeal, laryngeal, and esophageal cancer occurring within 2 months of screening were ascertained from the KDB. Generalized estimating equations with modified Poisson regression were used to estimate adjusted risk ratios (aRRs) comparing endoscopy with radiography.

**Results:**

Endoscopic screening was significantly associated with higher composite incidental detection rates for non-gastric upper aerodigestive tract cancers (95.9 vs. 34.3 per 100,000 examinations; aRR 2.94, 95% confidence interval [CI] 1.50–5.76; *p* = 0.002). Specifically, esophageal cancer detection was markedly higher with endoscopy (83.4 vs. 17.1 per 100,000; aRR 5.16, 95% CI 2.16–12.32; *p* < 0.001). No statistically significant differences were observed for oral cavity, pharyngeal, or laryngeal cancers.

**Conclusions:**

Endoscopic gastric cancer screening is associated with substantially higher incidental detection of non-gastric upper aerodigestive tract cancers, particularly esophageal cancer. These findings suggest an additional detection value for endoscopy that extends beyond its primary gastric cancer target in organized screening programs.

## Introduction

Gastric cancer remains a major contributor to cancer burden in East Asia [1, 2]. Historically, population-based gastric cancer screening in Japan, where organized municipal screening has been implemented for decades, primarily relied on radiographic photofluorography using an upper gastrointestinal barium contrast series (hereafter, “radiography”). In response to accumulating evidence supporting endoscopic screening, Japanese guidelines incorporated upper gastrointestinal endoscopy as an evidence-based modality for gastric cancer screening [3, 4].

Endoscopic screening generally provides superior performance for gastric cancer detection and has been supported by epidemiologic evidence suggesting a potential mortality benefit in real-world settings. Population-based studies from Japan have suggested an inverse association between endoscopic screening history and gastric cancer mortality [4], and large-scale evaluations from the Korean National Cancer Screening Program have shown lower gastric cancer mortality among individuals screened with endoscopy, whereas no comparable association has been observed for upper gastrointestinal radiography [5]. Importantly, upper gastrointestinal endoscopy traverses the upper aerodigestive tract and allows direct visualization from the nasal/oral cavity through the pharynx/larynx and esophagus to the stomach. This anatomical coverage provides an opportunity for incidental detection of malignancies such as oral cavity, pharyngeal/laryngeal, and esophageal cancers during a screening episode—an additional benefit not inherently expected with radiography. Procedure-based reports have described detection of pharyngeal cancers in screening endoscopy populations; however, population-based quantification of the incremental detection yield of endoscopy over radiography in routine municipal practice remains limited [6, 7].

If endoscopic screening contributes not only to gastric cancer detection but also to incidental identification of oral–pharyngeal–laryngeal–esophageal cancers, it may effectively function as a dual-purpose examination—gastric cancer screening plus opportunistic screening of the upper aerodigestive tract—and thus provide an additional rationale for selecting endoscopy over radiography in municipal programs. To examine this possibility, Okayama City’s municipal gastric cancer screening registry was linked with administrative claims data from the National Health Insurance database (Kokuho Database; KDB) [8, 9]. The aim of the study was to compare endoscopic screening with radiography and quantify detection of non-gastric upper aerodigestive tract cancers to assess the broader detection yield of endoscopic screening in routine population-based practice.

## Materials and methods

### Study design and setting

This retrospective cohort study was conducted using individually linked municipal gastric cancer screening records and health insurance claims data from the KDB in Okayama City, a prefectural capital in western Japan. Two administrative data sources were linked at the individual level: (1) the Okayama City municipal gastric cancer screening registry, which records screening dates and modalities (radiography or endoscopy); and (2) the KDB, an administrative database that includes monthly health insurance claims for beneficiaries of the National Health Insurance and the Latter-Stage Elderly Medical Care System, widely used for population-based epidemiologic research and insurer-based health management [8, 9]. According to the 2020 national census, the city population was 724,691 [10].

Residents who underwent municipal gastric cancer screening between April 2016 and March 2022 (fiscal years 2016–2021) were identified. Only individuals enrolled in the KDB who had a corresponding municipal gastric cancer screening record were included in the analytic cohort. Individuals aged < 50 years were excluded, as population-based gastric cancer screening in Japan targets individuals aged 50 years and older. Further exclusions included individuals with evidence of a prior diagnosis of any target cancer (oral cavity, pharynx, larynx, esophagus, or stomach) before their first eligible screening examination during the study period.

### Cancer screening in Japan

In Japan, cancer screening programs are broadly categorized into population-based (organized) screening, administered by municipalities, and opportunistic screening, offered at the discretion of individual healthcare or screening facilities. Population-based gastric cancer screening targets asymptomatic residents aged 50 years and older, recommended every two years per national guidelines [3]. Eligible residents receive a municipal subsidy that substantially reduces individual out-of-pocket cost. National guidelines recommend radiography and upper gastrointestinal endoscopy as evidence-based modalities which residents can choose whichever they prefer [3, 4].

Gastric cancer screening was designed with the primary programmatic goal of reducing related mortality. Accordingly, the program’s designated endpoint is gastric cancer, and program evaluation is structured around this outcome. Non-gastric upper aerodigestive tract cancer falls outside the designated target of the program. Since their detection is not the intended outcome of the program, reporting such incidentally detected malignancies to implementing bodies (municipalities) is not required under current program frameworks. As a result, population-level data on this potential additional benefit have been lacking [11].

### Exposure: screening modality

The exposure was the screening modality used in municipal gastric cancer screening: radiography or endoscopy. Radiography was performed to evaluate the stomach as the primary target organ of gastric cancer screening. In contrast, upper gastrointestinal endoscopy provides direct visualization along a continuous anatomical route—from the nasal/oral cavity through the pharynx/larynx and esophagus to the stomach.

### Outcomes: incidental cancer detection

The primary outcome was incidental detection of a composite of non-gastric upper aerodigestive tract cancers during a municipal gastric cancer screening episode. Cancer diagnoses were ascertained from the KDB using ICD-10 codes: oral cavity (C00–C06), pharyngeal (C09–C14), laryngeal (C32), and esophageal (C15) cancers [9]. The secondary outcome was incidental detection of each individual site assessed separately. For all primary and secondary outcomes, diagnoses occurring within 2 months of a screening examination were identified. The 2-month window was selected to allow sufficient time for the full diagnostic pathway, particularly for radiographic screening, in which an abnormal finding requires referral for subsequent confirmatory endoscopy before a definitive diagnosis can be made.

Additionally, gastric cancer (ICD-10: C16) detection rates and their association with screening modality were assessed as a supplementary reference analysis to contextualize the primary findings and to confirm the expected superiority of endoscopy for the designated target cancer; these results are presented alongside non-gastric outcomes.

### Statistical analysis

Participant characteristics were summarized by modality (radiography-only, endoscopy-only, and both modalities). Descriptive statistics included sex, age at the end of fiscal year 2021, and number of screening examinations by fiscal year and by total number of examinations per participant.

Incidental cancer detection was then characterized for each modality using three descriptive measures: number of cancers detected within the diagnostic window, detection rate per 100,000 screening examinations, and proportion of screened diagnoses among all individuals diagnosed with each cancer type during the study period (screened/total proportion).

To evaluate the association between screening modality and incidental cancer detection while accounting for within-person correlation arising from individuals contributing multiple screening examinations over the study period, generalized estimating equations (GEE) were applied with a modified Poisson regression model (log link function) and an exchangeable working correlation matrix, with each screening examination as the unit of analysis and resident identifier as the clustering unit. This approach estimates population-average effects across repeated screening episodes and is particularly suited for longitudinal data in which individuals contribute multiple observations over time. Robust (sandwich) variance estimators were used to obtain risk ratios (RRs) and 95% confidence intervals (CIs) [12]. Adjusted risk ratios (aRRs) were obtained by controlling for age at the end of fiscal year 2021 (continuous) and sex. Radiography served as the reference category; thus, aRRs greater than 1.0 indicate a higher detection rate with endoscopy. For outcome–modality combinations with sparse data (zero events in one modality), GEE estimates were not calculable, and results are reported as not calculable (NC).

A pre-specified sensitivity analysis was conducted to examine the primary and secondary outcomes using a 3-month diagnostic window, to assess robustness to the choice of diagnostic window; results are presented. All analyses were performed using Stata version 19 (StataCorp, College Station, TX, USA).

## Results

### Participant characteristics

**Fig. 1:**
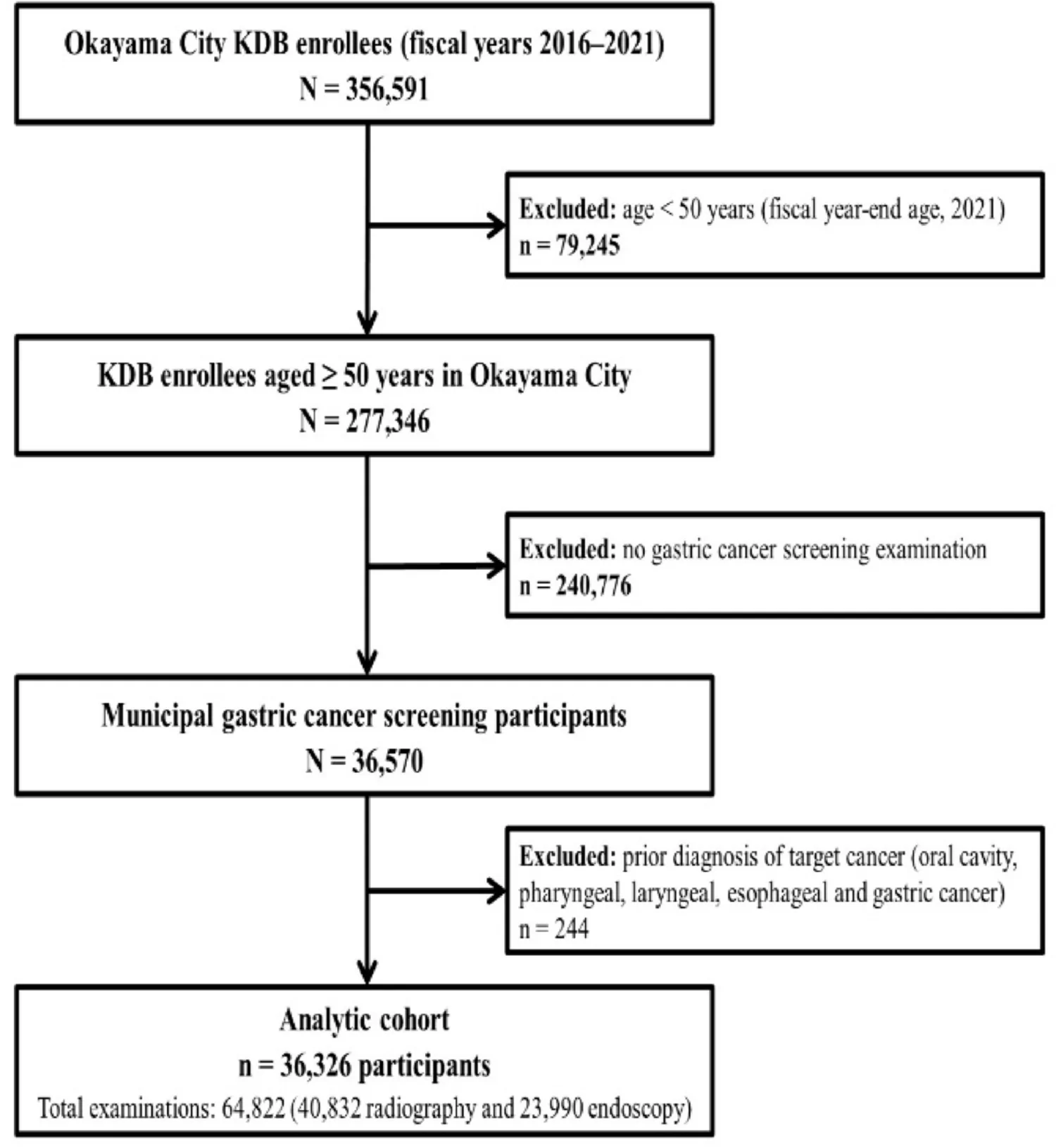
Flowchart of study participant selection KDB, Kokuho Database, *FY* fiscal year

A total of 36,326 participants who underwent municipal gastric cancer screening were included in the analytic cohort (Fig. 1), contributing 64,822 screening examinations (40,832 radiography; 23,990 endoscopy). Participant characteristics are summarized in Table 1.

**Table 1:** Participant and screening characteristics by modality group

Characteristic	Radiography (*n* = 21,227)	Endoscopy (*n* = 12,194)	Radiography and endoscopy (*n* = 2,905)
***Participant-level characteristics***			
Male sex, n (%)	8,995 (42.4)	4,851 (39.8)	1,189 (40.9)
Age at the end of FY 2021, median (IQR)	72 (66–76)	72 (67–76)	69 (65–74)
50–59 years, n (%)	1,686 (7.9)	804 (6.6)	229 (7.9)
60–69 years, n (%)	6,774 (31.9)	3,422 (28.1)	1,227 (42.2)
70–79 years, n (%)	9,329 (44.0)	6,109 (50.1)	1,198 (41.2)
80 years or older, n (%)	3,438 (16.2)	1,858 (15.2)	248 (8.5)
Number of examinations, median (IQR)	2 (1–2)	1 (1–2)	3 (2–3)
1 examination, n (%)	10,481 (49.4)	6,775 (55.6)	0 (0.0)
2 examinations, n (%)	5,898 (27.8)	3,150 (25.8)	1,315 (45.3)
3 or more examinations, n (%)	4,848 (22.8)	2,269 (18.6)	1,590 (54.7)
***Examination-level characteristics***			
Screening examinations, n (total)	37,031	20,225	7,566
FY 2016	7,810	3,971	1,468
FY 2017	7,411	3,214	1,229
FY 2018	6,543	3,370	1,337
FY 2019	5,713	3,562	1,253
FY 2020	4,855	2,888	1,069
FY 2021	4,699	3,220	1,210

Modality groups were defined based on a participant’s overall screening history during the study period (FY 2016–2021): radiography-only, endoscopy-only, or both modalities

*IQR* interquartile range, *FY* fiscal year (April to March)

### Primary outcome: non-gastric composite

Within the 2-month diagnostic window, the composite non-gastric upper aerodigestive tract cancer detection rate per 100,000 screening examinations was 34.3 for radiography and 95.9 for endoscopy. Of 201 individuals with a non-gastric upper aerodigestive tract cancer diagnosis during the study period, 37 (18.4%) received their diagnosis within 2 months of the screening examination (Table 2). After adjustment for age and sex, endoscopic screening was associated with a significantly higher incidental detection rate of the composite outcome (aRR 2.94, 95% CI 1.50–5.76) (Table 3).

**Table 2:** Incidental cancer detection events, rates per 100,000 examinations, and proportions of screened diagnoses by 2- and 3-month diagnostic windows

Outcome	2-month diagnostic window			3-month diagnostic window		
	Radiography events, *n* (per 100,000)	Endoscopy events, *n* (per 100,000)	Screened/Total, *n* (%)	Radiography events, *n* (per 100,000)	Endoscopy events, *n* (per 100,000)	Screened/Total, *n* (%)
***Primary outcome***						
Non-gastric composite	14 (34.3)	23 (95.9)	37/201 (18.4%)	19 (46.5)	27 (112.6)	46/201 (22.9%)
***Secondary outcomes***						
Esophageal cancer	7 (17.1)	20 (83.4)	27/90 (30.0%)	10 (24.5)	20 (83.4)	30/90 (33.3%)
Oral cavity cancer	3 (7.4)	1 (4.2)	4/77 (5.2%)	4 (9.8)	3 (12.5)	7/77 (9.1%)
Pharyngeal cancer	0 (0)	3 (12.5)	3/29 (10.3%)	2 (4.9)	3 (12.5)	5/29 (17.2%)
Laryngeal cancer	4 (9.8)	0 (0)	4/24 (16.7%)	5 (12.3)	2 (8.3)	7/24 (29.2%)
***Reference outcome***						
Gastric cancer	72 (176.3)	136 (566.9)	208/640 (32.5%)	88 (215.5)	146 (608.6)	234/640 (36.6%)

**Table 3:** Incidental cancer detection rate ratios for endoscopic versus radiographic screening by 2- and 3-month diagnostic windows

Outcome	2-month diagnostic window		3-month diagnostic window	
	Crude model RR (95% CI)	Adjusted model adjusted RR (95% CI)	Crude model RR (95% CI)	Adjusted model adjusted RR (95% CI)
***Primary outcome***				
Non-gastric composite	2.80 (1.44–5.43)	2.94 (1.50–5.76)	2.42 (1.35–4.35)	2.53 (1.40–4.57)
***Secondary outcomes***				
Esophageal cancer	4.86 (2.06–11.50)	5.16 (2.16–12.32)	3.40 (1.59–7.27)	3.61 (1.68–7.78)
Oral cavity cancer	0.57 (0.06–5.45)	0.57 (0.06–5.54)	1.28 (0.29–5.70)	1.24 (0.27–5.60)
Pharyngeal cancer	NC	NC	2.55 (0.43–15.28)	2.60 (0.43–15.68)
Laryngeal cancer	NC	NC	0.68 (0.13–3.51)	0.73 (0.14–3.75)
***Reference outcome***				
Gastric cancer	3.22 (2.42–4.28)	3.21 (2.41–4.28)	2.82 (2.17–3.68)	2.82 (2.16–3.68)

Adjusted model: adjusted for age and sex

*RR* risk ratio, *CI* confidence interval, *NC* not calculable

### Secondary outcomes: individual cancer sites

Detection rates and GEE estimates for individual cancer sites within the 2-month window are summarized in Tables 2 and 3. Of 90 individuals diagnosed with esophageal cancer, 27 (30.0%) were diagnosed within 2 months of screening, and endoscopic screening was associated with a markedly higher detection rate of esophageal cancer (radiography 17.1 vs. endoscopy 83.4 per 100,000; aRR 5.16, 95% CI 2.16–12.32). Pharyngeal cancer (3/29, 10.3%) was detected exclusively by endoscopy (0 radiography events vs. 3 endoscopy events; radiography 0 vs. endoscopy 12.5 per 100,000), and laryngeal cancer (4/24, 16.7%) exclusively by radiography (4 radiography events vs. 0 endoscopy events; radiography 9.8 vs. endoscopy 0 per 100,000); GEE estimates for both sites were NC owing to sparse data. Of 77 individuals with oral cavity cancer, 4 (5.2%) were diagnosed within 2 months of screening; detection was numerically higher with radiography (radiography 7.4 vs. endoscopy 4.2 per 100,000; aRR 0.57, 95% CI 0.06–5.54), but the difference was not statistically significant. As a supplementary reference, endoscopic screening was associated with substantially higher gastric cancer detection (208/640, 32.5%; radiography 176.3 vs. endoscopy 566.9 per 100,000; aRR 3.21, 95% CI 2.41–4.28; Tables 2 and 3), confirming the expected superiority of endoscopy for the program’s primary designated target cancer.

### Sensitivity analysis

Sensitivity analysis with a 3-month diagnostic window showed that event counts increased in both radiography and endoscopy across all sites; however, aRRs were largely consistent with the 2-month primary analysis, supporting the robustness of the findings (Tables 2 and 3). The additional events also enabled GEE estimation for pharyngeal cancer (aRR 2.60, 95% CI 0.43–15.68) and laryngeal cancer (aRR 0.73, 95% CI 0.14–3.75), both of which showed no statistically significant difference between modalities.

## Discussion

In this population-based retrospective cohort study linking municipal gastric cancer screening records with health insurance claims data in Okayama City, Japan, endoscopic screening was associated with significantly higher incidental detection rates of composite non-gastric upper aerodigestive tract outcomes compared with radiographic screening (aRR 2.94, 95% CI 1.50–5.76). Specifically, endoscopic screening was associated with a substantially higher detection rate of esophageal cancer (aRR 5.16, 95% CI 2.16–12.32). No statistically significant difference was observed in oral cavity cancer detection between modalities. These primary findings were robust to extension of the diagnostic window to 3 months.

Prior reports have described incidental detection of upper aerodigestive tract cancers during endoscopic gastric cancer screening. In Japan, Sato et al. identified two hypopharyngeal carcinomas and five esophageal carcinomas as incidental findings in a municipal gastric cancer screening program involving 9,142 endoscopic examinations, corresponding to a non-gastric upper aerodigestive tract cancer detection rate of 76.6 per 100,000 examinations [6]; a large-scale health checkup endoscopy study of 28,842 examinations by Kashiwagi et al. similarly detected six pharyngeal and 26 esophageal cancers, yielding a combined detection rate of 111 per 100,000 examinations [13]. In a Korean cohort of 2,720 endoscopic examinations, Yang et al. similarly reported five incidental upper aerodigestive tract cancers (183.8 per 100,000), including three pharyngeal, one laryngeal, and one oral cavity cancer [7]. In this study, the non-gastric composite detection rate with endoscopy (95.9 per 100,000 for the 2-month window; 112.6 per 100,000 for the 3-month window) falls within the range reported by these prior facility-based studies, suggesting consistency with prior reports and supporting the plausibility of our estimates.

The significantly higher esophageal cancer detection rate with endoscopy likely reflects the continuous and direct visualization of the esophageal mucosa inherent to upper gastrointestinal endoscopy. Because upper gastrointestinal endoscopy entails routine inspection of the esophageal mucosa, the esophagus is more likely to be systematically evaluated during endoscopic screening than during radiographic screening [14]. In Korea, participation in a national gastric cancer screening program was also associated with lower esophageal cancer-specific mortality, with a stronger association observed for upper endoscopy than for radiographic screening [15]. These findings may also have implications for risk-stratified screening. Gastric and esophageal squamous cell carcinomas share important etiological factors such as heavy alcohol consumption and tobacco use [16]. Therefore, endoscopic gastric cancer screening may also provide an opportunity for incidental detection of esophageal cancer among individuals at elevated esophageal cancer risk [17]. Endoscopic and radiographic screening may play different roles within organized gastric cancer screening programs. Endoscopy allows direct mucosal inspection and may lead to biopsy and diagnosis during the same examination, whereas radiographic screening has practical advantages for mass screening implementation. Accordingly, the present comparison should be interpreted in the context of real-world screening pathways rather than as a direct comparison of the intrinsic diagnostic performance of the two procedures alone. Importantly, our findings should not be interpreted as suggesting that radiographic screening is no longer warranted. Radiographic screening retains practical advantages in organized mass screening, including higher throughput capacity and feasibility in settings with limited endoscopic resources.

The absence of a statistically significant difference in oral cavity and pharyngo-laryngeal cancer detection between modalities should be interpreted cautiously, given the small number of site-specific events. In population-based programs, conscious sedation is generally not used owing to side effects, and the endoscope traverses the pharynx and larynx relatively rapidly [11]. Furthermore, the anatomical complexity of these regions and the absence of a standardized inspection protocol for pharyngeal and laryngeal sites in organized screening settings may limit the detection of malignancies at these anatomical sites. Additionally, oral cavity lesions are accessible for direct visual inspection during routine clinical encounters, such as dental or otolaryngologic consultation. Pharyngeal and laryngeal cancers frequently present with clinically apparent symptoms, including throat discomfort, dysphagia, or hoarseness, and are often diagnosed at locally advanced stage [18]. However, the incidental detection of esophageal cancer may prompt clinicians to recommend subsequent systematic evaluation of the pharynx and larynx, thereby potentially contributing to early detection of head and neck malignancies through a cascading clinical pathway [19]. Overall, endoscopic gastric cancer screening may offer an additional benefit for esophageal cancer detection, whereas its impact on oral and pharyngo-laryngeal cancer detection appears limited in current population-based practice.

This study has several notable strengths. First, to our knowledge, this is the first study to directly link a municipal gastric cancer screening registry with KDB administrative claims data to examine incidental non-gastric cancer detection. The KDB provides near-complete coverage of the target population for municipal gastric cancer screening, enabling ascertainment of cancer diagnoses regardless of where care was received. Second, this study provides the first population-based quantification of the incidental detection yield of endoscopic compared with radiographic gastric cancer screening for non-gastric upper aerodigestive tract cancers. Because such cancers are not the designated target of the screening program and their detection is not mandatorily reported to implementing bodies, they have been invisible to routine program evaluation; the present study directly addresses this evidence gap.

This study has several limitations. First, the 2-month diagnostic window involves an inherent trade-off between temporal specificity and sensitivity. Some cancers detected within this window—particularly at sites not directly visualized by radiography, such as the larynx—may not represent true screening-mediated incidental detections, potentially introducing nondifferential misclassification. However, as both modalities were evaluated within the same window, this limitation applies equally to both groups and does not invalidate the relative comparison. Second, event counts for individual cancer sites—particularly pharyngeal, laryngeal, and oral cavity cancers—were small, resulting in wide confidence intervals. Future studies with larger samples or multi-municipality data linkages are required for more precise site-specific estimates. Third, these findings may not be generalizable to opportunistic endoscopic screening settings, where conscious sedation and systematic pharyngeal inspection protocols are more commonly employed [11]. Fourth, screening modality was not randomly assigned but self-selected by participants. Prior Japanese studies suggest that individuals selecting endoscopy may differ modestly from those selecting radiography in demographic characteristics and health-seeking behaviors, which may have introduced residual confounding in the present study [20, 21]. Additionally, although population-based cancer screening is explicitly designed for asymptomatic residents, the possibility that individuals with unrecognized or mild symptoms preferentially selected endoscopy cannot be entirely excluded. Fifth, the analytic cohort was limited to participants linkable to the KDB. In addition, because cancer stage information was unavailable in the KDB, we could not directly assess whether cancers detected after screening were diagnosed at an earlier stage. Prior cancer history was ascertained from available KDB claims records before the first eligible screening examination; however, remote prior cancers may not have been fully captured if individuals had no relevant healthcare utilization after enrollment in the KDB. Additionally, detailed behavioral risk-factor data, such as smoking and alcohol consumption, were available only for a subset of participants with linked health checkup information; restricting analyses to that subset could have introduced substantial selection bias. Therefore, these factors were not included in the present analysis. Future studies with broader population coverage and more detailed clinical and behavioral data are needed.

In conclusion, endoscopic gastric cancer screening is associated with substantially higher incidental detection of non-gastric upper aerodigestive tract cancers, particularly esophageal cancer, compared with radiographic screening. These findings suggest that endoscopic gastric cancer screening may provide additional detection value beyond its primary gastric cancer target in organized screening programs. Future multi-municipality studies with larger sample sizes are needed to provide more precise estimates across individual cancer sites and to inform evidence-based program evaluation frameworks for population-based endoscopic gastric cancer screening.

## Data Availability

The data used in this study are derived from administrative databases held by Okayama City and the KDB and are not publicly available owing to data privacy regulations.
